# Diabetes podiatry services for Māori in Aotearoa: a step in the right direction?

**DOI:** 10.1186/s13047-022-00564-1

**Published:** 2022-08-09

**Authors:** B Ihaka, K Rome, H Came

**Affiliations:** 1 Faculty of Health & Environmental Sciences, School of Clinical Sciences, AUT, Auckland, 1142 New Zealand; 2 Faculty of Health & Environmental Sciences, School of Public Health, AUT, Auckland, 1142 New Zealand

**Keywords:** Indigenous, Māori, Diabetes, Amputation

## Abstract

Māori with diabetes are at a 65% greater risk of amputation compared to non-Māori with diabetes. Despite evidence to support the role of podiatrists in reducing diabetes-related lower limb amputations; the effectiveness of diabetes podiatry services at the community and secondary level to achieve this for Māori is largely unknown. Diabetes podiatry services need to be reorientated and be culturally applicable to Indigenous communities. Transforming diabetes podiatry services and practice may reduce Indigenous amputation rates and improve quality of life for an unserved community.

## Background

Globally, Indigenous people disproportionately carry the burden of disease, which constitutes a breach of the *Declaration on the Rights of Indigenous Peoples* [1]. Māori are the Indigenous people of Aotearoa. It is not certain where the title Māori came from (i.e., natives, ordinary) but *Māori* consider themselves the ‘tangata whenua’ the people of the land. Te Tiriti o Waitangi (Māori text) was negotiated by Māori and British Crown in 1840 and outlines the terms of British settlement. Te Tiriti affirmed Māori tino rangatiratanga (unfettered authority) and granted the Crown limited governorship over their (non-Māori) people. It also granted Māori the same rights as privileges as British subjects and recognised the importance of religious and cultural freedom. The subsequent breaches of Te Tiriti have had disastrous inter-generational effects on Māori health [2].

Several government inquiries have determined systemic ethnic health inequities [3]. A major theme of these reports is institutional racism and prejudice which are modifiable determinants of Māori health and wellbeing. Health care practitioners, including podiatrists are responsible for confronting and acting upon these issues [4]. The Health Practitioners Competency Assurance Act [2003] is the main driver for ensuring culturally competent and safe practices in recognition of Te Tiriti. This is a unique competency for podiatry registration in Aotearoa. The Podiatrist Board of New Zealand is the regulatory body operating who determine how this competency is promoted and reviewed. Podiatrists currently must demonstrate cultural competency in a 2-year cycle for a minimum of 3 h [4].

Māori philosophy around health is holistic with various models that capture no separation from the physical, social, emotional or spirituality of an individual or their whānau (extended family [5–8]. When these domains are in harmony, and Māori have full cultural autonomy, health and wellbeing improve.

Despite this worldview, the measurement of Māori wellbeing often centres on Western worldviews [9]. For Māori, “data encapsulates stories, kāranga (calling, invitation), whakairo (carvings), waiata (song) and the knowledge shared in wānanga (open discussion including reflection for collective decision-making)” and hence, measuring Māori wellbeing needs to be considered through a Māori lens [10]. Wellbeing measures need to incorporate Indigenous aspirations including cultural practices such as “land use, traditional livelihoods and customary activities, language and culture” [11]. The purpose of this commentary is to admit the need to improve diabetes podiatry services for Māori through Māori worldviews. Critical evaluation can determine if diabetes podiatry services are doing what is intended, and asks the question, “Is this the best way of doing things?” [12].

### Podiatry management of diabetes for Māori

Podiatrists are considered the gatekeepers of diabetic foot management in the prevention of lower limb amputations [13]. Diabetes-related lower limb amputation is preceded by diabetic foot ulceration which is the net effect of clinical and social variables [14, 15]. The podiatrist’s role is to review diabetes related symptoms and signs of which change the function and structure of the lower limb, through objective vascular and neurological assessment. This assessment allows the podiatrist to triage the threat of risk of ulceration and manage these consequences through means such as off-loading devices, self-management for health education in partnership with the person, or referral to secondary level (hospital based) podiatrists for ulcer management or surgical review [16]. However, unhelpful social and political factors that impinge on wellbeing of Indigenous populations include location of health services, infrastructure, educational and employment disadvantage; and cultural and language difficulties [17]. Focussing on biomedical risk factors alone as per the current triaging tools do little to curb the social injustices experienced by Indigenous people.

In Aotearoa, funding is available to podiatrists in the community including private practice to assess and manage people with diabetes or consider referral to secondary level podiatrists which is publicly funded. Ethnicity as an inclusion for diabetes podiatry services was based primarily on a sixfold relative risk of amputation as well as addressing the need for culturally appropriate and comprehensive services [18, 19]. However, since funded diabetes podiatry services have been available, Māori, pose a 65% greater risk of amputation compared to non-Māori, [15, 16].

These findings highlight a fragmented health system where the quality of the resources available differ nationally and regionally [20]. Furthermore, a recent report highlights inconsistency of the level of quality of diabetes services in Aotearoa [21]. One such concerning theme was limited podiatry and retinal screening services and inconsistent diabetes self-management education and support. Māori identified barriers and enablers to diabetes podiatry services over ten years ago, yet culturally appropriate services and resources are still inadequate [22]. More recent findings of systemic racism have led to a major health reform in Aotearoa, with the disestablishment of district health boards and the formation of a Māori Health Authority. There is an opportunity to transform current diabetes podiatry services to meet Māori aspirations and improve lower limb outcomes.

The emergence of Te Tiriti-led decolonising praxis is crucial to addressing the inter-generational trauma that Māori have endured for little under 200 years. A Critical Tiriti Analysis [23] of current services show less then favourable equity results, levels of self-determination, leadership for mutual benefit and cultural freedom. With this current knowledge and a restructure of the health sector, podiatrists aligned in this field will be challenged to improve clinical outcomes for Māori. Whilst indicated earlier that Māori health and wellbeing encompasses more than just the physical, podiatrists will have to rethink the socio-political factors that impinge on health outcomes of Māori. Podiatrists (Māori and non-Māori) will be able to access support through iwi (tribal), hapū (sub-tribe) and Māori communities to partner an approach to mitigate inequity and empower cultural autonomy. These collaborations will also be considered when informing cultural competency requirements by the Podiatrists Board of New Zealand [24].

In conclusion, despite support for diabetes podiatry in preventing lower limb amputations the effectiveness of these services is largely unknown for Māori. Given the current health reforms, evaluation of diabetes services for Māori through wānanga (open discussions) allows opportunity for engagement and collective decisions which is a better reflection of a Te Tiriti partnership.

## Data Availability

The data utilised in this commentary is readily accessible from an academic library.

## Abbreviations

AUT: Auckland University of Technology
PHO: Primary Health Organisation
